# The Use of Albuterol in Young Infants Hospitalized with Acute RSV Bronchiolitis

**DOI:** 10.1155/2012/585901

**Published:** 2012-08-26

**Authors:** Michael T. Del Vecchio, Laura E. Doerr, John P. Gaughan

**Affiliations:** ^1^Department of Pediatrics, Temple University School of Medicine, Philadelphia, PA 19140, USA; ^2^Department of Pediatrics, Emory University, Atlanta, GA 30322, USA; ^3^Department of Epidemiology and Biostatistics, Temple University School of Medicine, Philadelphia, PA 19140, USA

## Abstract

*Objective*. To evaluate the effects of albuterol use in young infants admitted with respiratory syncytial virus (RSV) bronchiolitis with regards to length of time on supplemental oxygen and length of stay (LOS). To consider the possibility that albuterol use may increase the need for supplemental oxygen and increase LOS. *Design, Setting, and Participants*. Full-term infants between the ages of 11 days and 90 days (*N* = 316) were included in this retrospective study. Infants included were hospitalized with a diagnosis of RSV bronchiolitis at a university-affiliated children's hospital. *Results*. In 4 of 5 severity groups, patients who received albuterol required more time on supplemental oxygen and had longer LOS. The differences only reached statistical significance in one of the severity groups in regards to LOS. *Conclusions*. The use of albuterol does not appear to be useful in the treatment of young infants with RSV bronchiolitis and may actually be harmful, in regards to increased supplemental oxygen need.

## 1. Introduction

 Respiratory syncytial virus (RSV) is the leading cause of lower respiratory tract infections such as bronchiolitis and pneumonia in pediatric patients [1]. RSV bronchiolitis affects infants between the ages of 0–3 months with greater severity [2]. The viral process impairs normal pulmonary gas exchange leading to ventilation-perfusion (*V*/*Q*) mismatch and subsequent hypoxemia.

The care of infants with bronchiolitis is mostly supportive. The focus of therapy is providing supplemental oxygen as needed and intervening in regards to the infant's hydration status, if feeding is impaired. Bronchodilators have been extensively studied to determine if they are useful in the management of RSV bronchiolitis, with inconclusive results [2–4]. Bronchodilators continue to be used frequently in outpatient settings, for which there is some supportive data [5]. Far less data has been available in regards to the use of albuterol in the inpatient setting. Wainwright et al. looked at the use of bronchodilators in admitted patients with bronchiolitis and found no significant difference in regards to LOS or hours on oxygen when comparing epinephrine use to placebo [6]. In addition to the lack of data regarding use of bronchodilators for admitted patients with bronchiolitis, the wide variability of ages used in studies has led to confounding evidence.

Given the limitations of the available data addressing the use of bronchodilators for treatment of young infants admitted to the hospital with RSV bronchiolitis we undertook the present study. We aimed to evaluate whether young infants treated with albuterol had an increased duration of supplemental oxygen needs when compared to young infants who did not receive albuterol. We proposed that young infants treated with albuterol would be more likely to incur *V*/*Q* mismatch, leading to hypoxemia as measured by pulse oximetry, and increased length of time on supplemental oxygen. We also thought that an increase in length of time on supplemental oxygen would lead to an increased LOS. 

## 2. Methods

 We conducted a retrospective study during a 5-year period (1999–2004) by reviewing medical records of all infants between the ages of 11 to 90 days admitted to the general pediatric service at Temple University Children's Medical Center in Philadelphia, Pennsylvania, USA. Inclusion criteria included full-term infants (>37 weeks gestation), RSV antigen positive by the rapid RSV test, and infants from 11 to 90 days of age. Our exclusion criteria for the study were prematurity, a history of congenital cardiac or respiratory anomalies, use of corticosteroids during the course of hospitalization, other comorbidities which could affect respiratory status such as croup, pertussis, influenza, and asthma, or a transfer to the intensive care unit during the course of hospitalization.

### 2.1. Development of the Severity Score

 We developed a severity score based on the clinical symptoms reported by health care providers at our institution (Table 1). Given the retrospective nature of this study, it was critical to rely mostly on objective measurements. We therefore weighted the pulse oximetry measurements and the respiratory rate more heavily than the subjective measurements, such as auscultative findings and degree of respiratory distress, in calculating a score for each patient.

### 2.2. Medical Record Abstractions

 Internal Review Board approval was obtained before any data abstraction was undertaken. A standard form was used which included the following: age (in days), gestation (in weeks), birth weight, sex, race, hours on oxygen supplementation, number of doses of albuterol administered both in the emergency room and upon admission to the inpatient floor, length of hospitalization (in days), and bronchiolitis severity score, obtained from admission data. Data was entered into an Excel spreadsheet and checked for completeness and elimination of those who met exclusion criteria by reviewer (LD).

### 2.3. Statistical Methods

 Data from the medical records were analyzed using a two-way (treatment severity) analysis of variance. The null hypothesis was that there would be no difference between treatments or severities. Prior to analysis, all data were tested for normality using the Shapiro-Wilk test. The data were significantly nonnormal for all variables. In order to apply ANOVA methods, a “normalized-rank” transformation was applied to the data. The rank-transformed data was analyzed using a GLM ANOVA followed by multiple comparisons to detect significant mean differences between subgroups based on treatments and severities. Multiple pair-wise comparisons used the Bonferroni adjustment to maintain an experiment-wise type I error of 0.05 or less. Differences between means (rejection of the null hypothesis) were considered significant if the probability of chance occurrence was ≤0.05 using two-tailed tests.

## 3. Results

**Figure 1:**
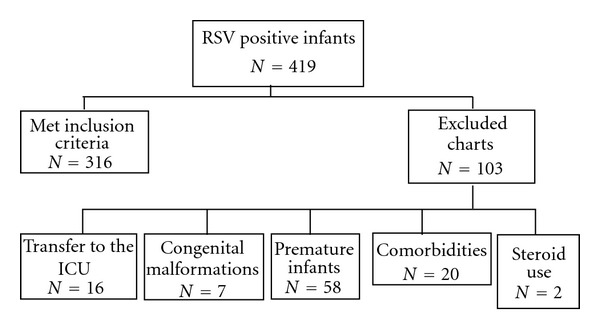
Selection of infants based on inclusion and exclusion criteria.

 A total of 419 patients were reviewed (Figure 1). Of the total, 103 patients were excluded leaving 316 patients for analysis. 56% were male. Average LOS was 2.47 days.

In all severity groups except for one the mean number of hours on supplemental oxygen was longer for the albuterol groups versus the nonalbuterol groups (Table 2). For the five severity groups which showed longer need of supplemental oxygen in the albuterol groups the increase in hours ranged from 3.4 to 21.6 (Table 2). None of the differences in the severity groups in regards to hours of supplemental oxygen reached statistical significance. In one of the severity groups the albuterol group had a shorter time of supplemental oxygen than the non-albuterol group (Table 2).

 In all severity groups except for one the LOS was longer in the albuterol groups than in the non-albuterol groups (Table 3). The increase in LOS for the severity groups receiving albuterol ranged from 0.41 days to 1.14 days. One of the groups reached statistical significance in regards to the increased LOS in the albuterol group. One severity group had a longer LOS in the non-albuterol group. This increase, though, was only 0.04 days.

 When all groups were taken together, statistical significance was not reached.

## 4. Discussion

 The use of bronchodilators in the treatment of bronchiolitis remains controversial. In spite of more evidence showing that bronchodilators are not helpful [6, 7], they continue to be used in the hope that they might help and that they do not cause harm. Additionally, there is little data to guide the use or nonuse of albuterol in the youngest of patients admitted with RSV bronchiolitis. A recent meta-analysis failed to find evidence for steroid or bronchodilator use in the inpatient setting [8]. We postulated that albuterol might actually be harmful by causing *V*/*Q* mismatching leading to a prolonged need of supplemental oxygen and subsequent LOS. In the present study we attempted to alleviate some of the potential confounding variables found in prior studies. First, we chose only young infants, 11–90 days of age. Second, we chose only patients who were RSV positive. Though it is not clear if there is a marked difference in the course of bronchiolitis depending on the viral pathogen, we chose to make our population as homogenous as possible. As in the study by Wainwright [6], we have shown that bronchodilators, albuterol in the present study, do not reduce the length of time that supplemental oxygen is required or LOS. However, we have additionally shown that in young infants with RSV bronchiolitis, albuterol may actually prolong the time that supplemental oxygen is required and lead to increased LOS. The end point of LOS has many factors involved in its final analysis. One is clinician and parent level of comfort for care at home. If a patient is receiving a specific intervention, it may increase the anxiety level of the caregiver and thus increase the LOS. As our results show, the LOS was even more prolonged than could be answered by the time of required supplemental oxygen. Though our results did not reach statistical significance in all but one group, the trends are clear. The one severity group (group 4) that did not show an increase time on supplemental oxygen and LOS in the albuterol group verses the non-albuterol group had an unexplainable low number of patients in the non-albuterol group (Tables 2 and 3) and this may have introduced error.

 Several limitations to the present study are worthy of consideration. First, given the retrospective nature of the study many variables were not available to take into consideration. For example, extensive demographic information could not be reliably extracted from the patient records. Thus we chose to compare patients in regards to a severity score. Additionally, we attempted to evaluate a homogenous population in regards to age and infecting agent. Secondly, though our overall number of patients was robust, some of the individual severity groups were quite small, which could certainly introduce error in our results. Lastly, though statistical significance was not reached, we do believe that the trends noted can be useful to the clinician caring for this patient population. Future inpatient studies would need to be large and prospective. It would also be critical that they should evaluate as homogenous a population as possible in regards to age and illness.

## 5. Conclusion

 Though use of bronchodilators in bronchiolitis remains controversial, most of the evidence addresses the lack of response, not possible negative outcomes. The present study adds to the existing evidence that, for young infants with RSV bronchiolitis admitted to the hospital, albuterol use is not helpful. Additionally, we have shown that bronchodilators may actually be harmful, leading to increase in time needed on supplemental oxygen and increase in LOS.

## Figures and Tables

**Table 1:** Bronchiolitis severity score.

Assessment measure	Numerical score
	(0) <50
Respiratory rate	(1) 50–70
	(2) >70

Accessory muscle use	(0) Normal
	(1) Use of any accessory muscles

	(0) RA saturation ≥95%
Oxygen saturation	(1) RA saturation 90–94%
	(2) RA saturation <90%

Auscultation	(0) Clear
	(1) Noisy

Air entry	(0) Normal
	(1) Impaired

**Table 2:** Albuterol use, severity, and hours on oxygen.

Albuterol use	Severity	*N*	Hours on oxygen	SD	Difference in hours	*P* value
No	0	29	03.483	10.679		
Yes	0	7	09.143	15.625	5.660	>.9999
No	1	58	13.310	31.044		
Yes	1	29	16.724	42.028	3.414	>.9999
No	2	38	14.184	51.817		
Yes	2	49	35.796	52.554	21.612	0.1296
No	3	17	21.471	34.814		
Yes	3	51	27.647	44.356	6.176	>.9999
No	4	7	47.571	49.725		
Yes	4	19	38.316	51.448	−9.255	>.9999
No	5	4	60.000	63.251		
Yes	5	6	64.500	81.099	4.500	>.9999

**Table 3:** Albuterol use, severity, and length of stay.

Albuterol use	Severity	*N*	Length of stay	SD	Difference	*P* value
No	0	29	1.7931	0.9016		
Yes	0	7	2.2857	1.1127	0.4926	>.9999
No	1	58	2.1724	1.6664		
Yes	1	29	2.5862	1.9733	0.4138	>.9999
No	2	38	1.9737	2.0466		
Yes	2	49	3.1224	2.1078	1.1487	0.0294
No	3	17	2.1765	1.2367		
Yes	3	51	2.7059	1.8253	0.5294	>.9999
No	4	7	3.1429	1.8645		
Yes	4	20	3.1000	2.4688	−0.0429	>.9999
No	5	4	3.7500	2.3629		
Yes	5	6	4.6667	3.3862	0.9167	>.9999
